# A Dynamic Lane-Changing Driving Strategy for CAV in Diverging Areas Based on MPC System

**DOI:** 10.3390/s23020559

**Published:** 2023-01-04

**Authors:** Hongben Liu, Xianghui Song, Bo Liu, Jia Liu, Huan Gao, Yunyi Liang

**Affiliations:** 1 Research Institute of Highway Ministry of Transport, Beijing 100088, China; 2 Department of Automation, Tsinghua University, Beijing 100083, China; 3 School of Transportation Engineering, Central South University, Changsha 410017, China

**Keywords:** connected and automated vehicle (CAV), freeway diverging area, mixed traffic flow, optimal lane change driving strategy, model prediction control (MPC), moving horizon optimization

## Abstract

Freeway-diverging areas are prone to low traffic efficiency, congestion, and frequent accidents. Because of the fluctuation of the surrounding traffic flow distribution, the individual decision-making of vehicles in diverging areas is typically unable to plan a departure trajectory that balances safety and efficiency well. Consequently, it is critical that vehicles in freeway-diverging regions develop a lane-changing driving strategy that strives to improve both the safety and efficiency of divergence areas. For CAV leaving the diverging area, this study suggested a full-time horizon optimum solution. Since it is a dynamic strategy, an MPC system based on rolling time horizon optimization was constructed as the primary algorithm of the strategy. A simulation experiment was created to verify the viability of the proposed methodology based on a mixed-flow environment. The results show that, in comparison with the feasible strategies exiting to off-ramp, the proposed strategy can take over 60% reduction in lost time traveling through a diverging area under the premise of safety and comfort without playing a negative impact on the surrounding traffic flow. Thus, the MPC system designed for the subject vehicle is capable of performing an optimal driving strategy in diverging areas within the full-time and space horizon.

## 1. Introduction

Traffic problems have been recognized as one of the vital factors hindering economic development, with the impact of transportation on economic development becoming increasingly prominent. Data from the National Highway Traffic Safety Administration (NHTSA) show that driver causes were to blame for 93% of traffic accidents, while lane-changing was responsible for 27% of all incidents. The results of the China Field Operational Tests (China-FOT) also revealed that 23.91% of all incidents were dangerous accidents brought on by lane-changing [1]. Some empirical research has proved that lane-changing maneuver is one of the main triggers of traffic congestion in diverging areas [2,3,4]. Vehicles in diverging areas tend to decelerate to reach the speed limit of the off-ramps and change lanes to complete the driving task. On account of higher competition for space by frequent lane-changing behaviors and stronger fluctuation of traffic flow compared with other sections along the freeway, diverging areas have become critical bottleneck sections of freeways compared with other sections along the freeway. Subject to these typical sophisticated characteristics, freeway-diverging areas are prone to low traffic efficiency, congestion, and frequent accidents [5,6]. Consequently, it is imperative for vehicles in freeway-diverging areas to propose a lane-changing driving strategy that aims to improve the safety and efficiency of diverging areas simultaneously.

In recent years, with the technical maturity of Vehicle to Everything (V2X) communication and the comprehensive promotion of road infrastructure, the emergence of Connected and Autonomous Vehicles (CAVs) provides an opportunity to address the aforementioned problems. The Roadside Units (RSU) may characterize the immediate surroundings using data gathered in real-time by radars, cameras, GPS, and other devices. CAVs utilize V2X wireless communication and vehicle automation to enable systematic planning and coordinated control for vehicular operations. There would be significant improvements in response time, situational awareness, decision-making, planning, and operational execution for CAVs, which would lead to improved longitudinal and lateral motion performance [7,8]. In comparison with Human-driven Vehicles (HDVs), traffic flow consisting of CAVs is more inclined to achieve greater system-wide benefits in safety, stability, and efficiency [9].

For the purpose of improving the efficiency and safety of diverging areas, two primary aspects should be concentrated specifically: (1) vehicle operational features and interaction behaviors, which are microscopic vehicle characteristics such as car-following, lane-changing, collision avoidance, and so on; (2) macroscopic driving strategies in diverging under complex environment [10], which is a path planning problem in complex scenarios. Related research based on V2X technology has already been conducted in these two aspects to solve the aforementioned problems.

On the one hand, the studies on vehicle microscopic characteristics aim to assist drivers and improve driving safety, with the adaptive cruise control (ACC) system as one of the most popular CAV technologies developed to date. Previous theoretical research and empirical simulations have been extensively conducted to demonstrate the efficiency and stability of the ACC system [11,12]. The major focuses of the ACC system are the control strategies for individual CAVs and platooning of CAVs based on their impact on traffic flow characteristics. Particularly, the California Program on Advanced Technology for the Highway (PATH) has conducted long-term studies on the ACC system, and the proposed model validation has been certificated through practical vehicle experiments [13,14,15].

However, compared to the car-following works, the lane-changing maneuver is more complicated from the perspective of vehicle control, leading to more limitations. In early studies, Gipps et al. [16] pioneered the introduction of the most basic lane-changing decision model by analyzing the actual driving behaviors of drivers, and subsequent scholars have made a lot of improvements and extensions on this basis. In recent years, researchers have been more dedicated to developing an autonomous lane-changing model that is appropriate for CAV. The existing studies have been conducted around lane-changing decisions and trajectory planning and tracking.

As to lane-changing decision-making, rule-based and machine-learning methods are the two main different methods. Firstly, rule-based methods generally create lane-change decision models that resemble human drivers and have established parameters that would modify the algorithm for a certain environment. For example, a lane-changing decision model for CAVs with a two-level threshold that conforms to a driver’s perception of the ability to safely change lanes with a rear vehicle approaching fast was established [17]. This method has the advantage of fully considering the various risk perception of different drivers and utilizing real lane-changing data. Nevertheless, several methods based on deep reinforcement (RL) learning had been proven that the RL model has a superior capability in dealing with uncertain and stochastic environments compared with the rule-based baseline methods [18,19,20]. Additionally, a harmonious lane-changing decision-model-based RL [21] was designed to balance overall, and individual efficiency relied on individual sensing results. Furthermore, a case study by Dong et al. [22] integrated data gathered by other vehicles with sensing and networking capabilities that were nearby the CAV as well as those that were farther downstream to guide lane-changing.

The trajectory planning and tracking is to prevent possible crashes during the lane change procedure and maintain tracking performance. The trajectory planning approach by Luo et al. [23] converts the planning issue into a constrained optimization problem by using the lane change time and distance. Potential collisions during the lane change process can be effectively avoided throughout the lane change procedure using the suggested dynamic automated lane change maneuver. On account of dynamic collision avoidance, the prediction of the motion state of the surrounding vehicles is the critical part of this module. Then, the real-time motion prediction modules, which can handle intricate lane change scenarios for CAVs trajectory planning, were established, such as a combination of the probabilistic and deterministic prediction method and machine learning-based lane change intention prediction method [24]. The aforementioned two methods can evaluate a vehicle’s intention to change lanes within a brief period of time. However, this research on vehicle motion prediction mostly examined human intention mechanisms in the beginning to obtain a holistic grasp of driver intention [25].

The methods suggested in the aforementioned study have the benefit of considering all of the vehicle’s microscopic characteristics, which can assure both the safety and the precision of the trajectory tracking under conditions of especially low traffic density. However, the underlying environments of most of these studies are microscopic local scenarios with low traffic volume, single traffic flow composition, and simplified characteristics. These methods cannot adapt the feasibility analysis of lane-changing when the traffic volume is significant in such a manner that the lane-change process ignores the changing features of the lane-change gap.

On the other hand, the macroscopic driving strategy can improve the maneuverability and safety of traffic from the external environment [26,27,28]. This study refers to the strategy of the vehicle from entering the diverging impact area to traveling to the off-ramp, similar to the car local path planning. However, substantial attention has been dedicated to the fundamental parts of roads among the existing studies, whereas strategies designed specifically for ramps or emergencies are exceedingly sparse, particularly in diverging areas [29,30].

The traditional management strategy generally improved the efficiency and safety of diverging areas by using lane assignment strategy and signal control at the off-ramp [31,32], which could require vehicles to change lanes only where allowed. The suggested strategies demonstrated that allocating multiple lanes to exit traffic can alleviate congestion from affected upstream ramps and reduce interference between weaving and non-weaving traffic streams [33,34]. However, the placement of traffic markings and barriers is essential for this strategy, which may impose some constraints, such as the flexibility of the management plan and the reaction of drivers.

Hence, more consideration has been given to the lane-changing driving strategy around the vehicle itself in the diverging area over the years. Dong et al. [10] proposed a route control strategy for driving through off-ramps and determined an optimal control grading strategy for traffic congestion dissipation and travel delay reduction among the proposed three route control strategies. It is demonstrated that the cooperative lane-changing strategy can improve traffic operation, traffic safety, and traffic oscillation in diverging areas [35,36]. Furthermore, Tajalli [37] established a cooperative distributed algorithm, which enables CAVs to formulate their optimal trajectories based on predicted information from surrounding vehicles and coordinate their lane-changing decisions to avoid collisions. However, in a mixed-traffic environment, these strategies are regarded as ego-efficient since they prioritize the interests of CAVs over those of surrounding vehicles. With the growth of the CAV population, the ego-efficient lane-changing strategies would probably lead to the renowned “Tragedy of the Commons” due to road resources are limited [38].

In summary, it is essential for CAVs to establish a method to deal with microscopic characteristics in intricate traffic scenarios, especially in diverging areas which involve multiple vehicles changing lanes multiple times. The effectiveness of automated lane-changing for CAVs when interacting with HDVs needs to be further investigated. The majority of studies on macroscopic driving strategy only focus on the vehicle motion state in the local area around CAV and lack the perception and prediction of the traffic flow movement state in full time and space horizon, as a result of failing to formulate a global optimal strategy.

Based on the complex scenario of diverging areas, this study proposed a full-time horizon optimal strategy for CAVs exiting the diverging area. Since it is a dynamic strategy, an MPC system based on rolling time horizon optimization was designed as the fundamental algorithm of the strategy. The major contributions of this study are summarized as follows:

(1) The study proposed a rolling state prediction model that can be applied to both CAVs and HDVs and can make rolling predictions in the full-time horizon after the *SV* enters the diverging area. The rolling prediction is characterized by the ability to update the prediction weighting factor and error factor in real-time to ensure the accurate prediction of different types of vehicles’ motion states.
(2) The development of the driving strategy proposed is based on the state information of all vehicles in the diverging area in the full-time horizon, with the ability to achieve the optimal global strategy in terms of efficiency. Simultaneously, it can ensure the safety and comfort of the vehicle throughout the driving process of the *SV*. Compared to existing studies, the strategy takes the overall traffic operation efficiency of the diverging area into account by designing an evaluation function while focusing on the self-efficiency of the *SV*.

The remainder of this paper is organized as follows: Section 2 describes the methods of the algorithm proposed in the manuscript. Section 3 designs a simulation experiment to demonstrate the validation of the strategy. Section 4 presents a numerical analysis of experiment results, followed by concluding remarks in Section 5.

## 2. Methods

### 2.1. An Overview

The study focus is the CAVs that must depart the freeway in a situation with mixed traffic flow, and the vehicle is referred to as the subject vehicle (*SV*). A dynamic lane change driving strategy is suggested in the diverging area scenario to tackle the issues with traffic safety and efficiency. This approach aims to make it possible for *SV* to change lanes and exit the freeway as efficiently as possible. In this study, in order to examine the advantages brought about by the model approach, the diverging area is divided into two parts according to the following guidelines:

(1) Discretionary Lane-Changing Area (DLA): With the analysis of actual freeway driving data, we take the location where the lane change behavior occurs upstream of the diverging area ramp as the starting point, and the starting point of the off-ramp as the endpoint, which is the region $P_{1}\left(L_{1},\;L_{2}\right)$. The vehicles in this region can freely change lanes according to the driving strategy.

(2) Mandatory Lane-Changing Area (MLA): We take the starting and end point of the off-ramp as the starting and end point of this area the starting point of the off-ramp as the endpoint, which is the region $P_{2}\left(L_{2},\;L_{3}\right)$. If there is an insertable gap in the target lane (TL) in this area, a lane-changing action is made immediately to complete the travel intention.

**Figure 1:**
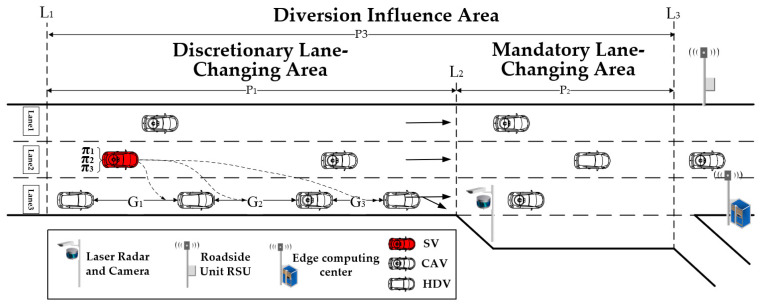
The schematic of the diverging influence area.

We define the region $P_{3}\left(L_{1},\;L_{3}\right)$ as diverging impact area (DIA). The traffic flow in this region is disturbed due to the influence of the subject vehicle lane-changing, which leads to the disturbance of the traffic flow characteristics, and the region designed for this model to analyze the traffic flow efficiency. The schematic of the diverging influence area can be shown in Figure 1.

In order to establish a complete dynamic lane change strategy in the diverging area, we need to have a full comprehension of the traffic flow operation, so the following assumptions about the mainline traffic and infrastructure in the diverging area are made:

(1) Mainline vehicles without departure do not take lane change behavior, and the HDVs with lane change demand would make lane change before entering DIA and keep straight until they accomplish the travel task.
(2) Mainline vehicles in TL would not generate insertable gaps for *SV* cooperatively.
(3) *SV* is capable of sensing complete information on vehicle motion state in the diverging area with the contribution of V2X technology.

**Figure 2:**
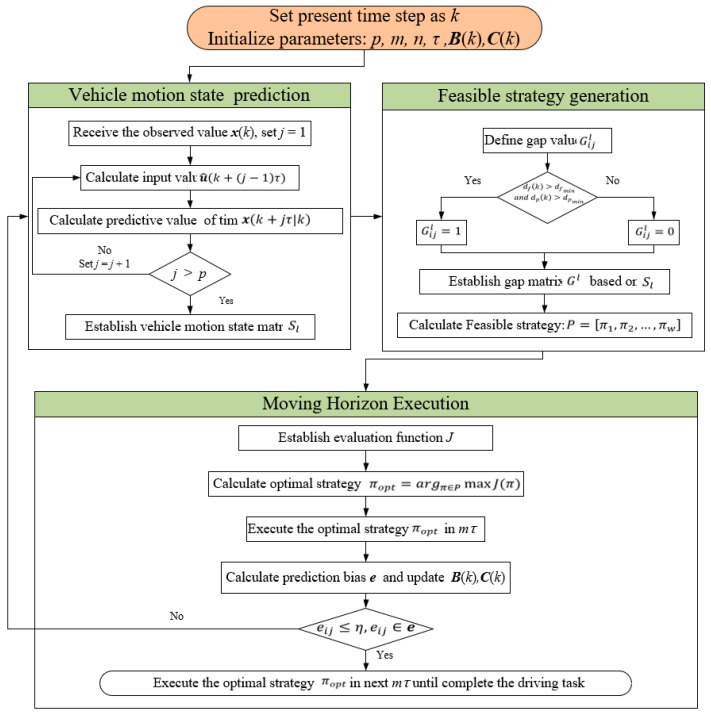
Methodology framework.

The Methodology framework can be seen in Figure 2. Nomenclatures used in this section are described in Table A1 of the Appendix A.

### 2.2. Vehicles Motion State

A vehicle in motion consists of movements in two dimensions: longitudinal and lateral movements. Longitudinal motion is mainly reflected in maintaining the desired velocity and keeping a safe distance from the preceding vehicle (i.e., following behavior). The lateral motion is mainly reflected in whether the vehicle makes a lane change action (overtaking, departing away, merging, etc.).

Numerous related investigations on the longitudinal and lateral motions of vehicles have been carried out. The following is one of the primary categories for the research of the car-following model: (1) Stimulus-Response model such as GM [39]. (2) Safe distance model such as Gipps model [40]. (3) Optimal Velocity model such as OVM, Newell model, FVD [41,42]. (4) Intelligent Driver Model (IDM) [43,44]. (5) Adaptive Cruise Control (ACC) and its more sophisticated variant Cooperative ACC (CACC), developed by the PATH laboratory of the University of California at Berkeley [45]. For CAVs, commonly used lane change trajectory planning models mainly include models based on Search Algorithms [46], Artificial Potential Field [47], and Parameterized Curves [48].

In this study, the IDM, ACC, and CACC are used to capture car-following behaviors of HDVs, CAVs, and platoon of CAVs, respectively. As to trajectory planning algorithms, the fifth-degree polynomial algorithm is used to compute the trajectory of a vehicle changing lanes in this model since that offers the advantages of high order differentiability of the trajectory equation, fewer points required to determine the trajectory, and flexibility of dynamic adjustment.

#### 2.2.1. Longitudinal Action

The Intelligent Driver Model (IDM) was proposed by Treiber et al. [43]. The following behavior of the human-driven vehicle is expressed using this model because it effectively simulates the psychological state of the driver. The model is shown as follows:

(1) $$\left\{\begin{array}{c} a_{i}=a_{m}\left[1-{\left(\frac{v}{v_{d}}\right)}^{\delta }-{\left(\frac{s^{*}}{s}\right)}^{2}\right]\\ s^{*}=s_{0}+vT+\frac{v_{i}∆v}{2\sqrt{a_{m}b}}\;\;\;\;\;\;\;\;\;\;\;\;\; \end{array}\right.,\;i=1,2,\ldots ,n$$

where $a_{i}$ is the acceleration of the vehicle; $a_{m}$ is the maximum acceleration;$\;v_{d}$ is the desired velocity; δ is the free acceleration exponent; $s^{*}$ is the expected distance from the preceding vehicle; s is the actual distance from the preceding vehicle; $s_{0}$ is the static safe distance; T is the desired time headway; b is the desired deceleration; ∆v is the velocity difference from the preceding vehicle.

The PATH laboratory of the University of California at Berkeley proposed the ACC and CACC model to capture the car-following behaviors of CAVs. Several actual vehicle tests in miniature have been conducted to demonstrate the model car-following performance. On this basis, the ACC and CACC model are adopted in this study to capture the behaviors of a single CAV and a platoon of CAVs. The ACC model is written as:

(2) $$\left\{\begin{array}{c} e=∆x-vt_{a}-L-s_{0}\\ a_{i}=k_{1}e+k_{2}∆v\;\;\;\;\;\;\;\;\;\;\; \end{array}\right.$$

where ∆x is the space headway; $t_{a}$ is the time headway of ACC vehicles; L is the length of the vehicle; ∆v is the velocity difference between preceding vehicle and subject vehicle; e is the space headway error between the actual distance and the desired distance; $k_{1}$ and $k_{2}$ are the space headway error control parameters and velocity difference control parameters, respectively.

The CACC model is written as:

(3) $$\left\{\begin{array}{c} e=∆x-vt_{c}-L-s_{0}\;\;\;\;\;\;\;\;\;\\ a_{i}=v_{p}+k_{1}e+k_{3}\dot{e}\;\;\;\;\;\;\;\;\;\;\;\;\; \end{array}\right.$$

where $t_{c}$ is the time headway of CACC vehicles; $v_{p}$ is the velocity of the subject vehicle at the previous time step; $\dot{e}$ is the derivative form of space headway error; $k_{3}$ is the control parameter of $\dot{e}$.

#### 2.2.2. Lateral Action

Lateral action has a crucial impact on the safety and efficiency of diverging area. Thus, to be more convinced, this study selected a suitable trajectory function to model the lane-changing action. Here we choose the fifth-degree polynomial model based on time, which has the advantages of a closed form, a continuous third derivative and smooth curvature. The format of the reference trajectory is written as follows.

(4) $$\left\{\begin{array}{c} x\left(t\right)=c_{5}t^{5}+c_{4}t^{4}+c_{3}t^{3}+c_{5}t^{2}+c_{1}t^{1}+c_{0}\;\;\;\;\\ y\left(t\right)=d_{5}t^{5}+d_{4}t^{4}+d_{3}t^{3}+d_{5}t^{2}+d_{1}t^{1}+d_{0} \end{array}\right.$$

where $x\left(t\right)$ and $y\left(t\right)$ are the longitudinal and lateral distance at time t respectively, $c_{0}$ to $c_{5}$ and $d_{0}$ to $d_{5}$ are coefficients parameters. Then the coefficients matrixes can be defined as follows:

(5) $$C=\left[\begin{array}{cccccc} c_{5} & c_{4} & c_{3} & c_{2} & c_{1} & c_{0} \end{array}\right]\;\;\;\;\;D=\left[\begin{array}{cccccc} d_{5} & d_{4} & d_{3} & d_{2} & d_{1} & d_{0} \end{array}\right]$$

Twelve unknown coefficients can be calculated by the boundary conditions of the lane change process as follows:

(6) $$g_{h}=g\left(t_{h}\right),\;\forall g\in \left(x,\dot{x},\ddot{x,}y,\dot{y},\ddot{y}\right)\&h\in \left(0,f\right)$$

where $x_{0}$ and $y_{0}$ are the current longitudinal and lateral position, respectively, ${\dot{x}}_{0}$ and ${\dot{y}}_{0}$ are the current longitudinal and lateral velocity, respectively, ${\overset{˙˙}{x}}_{0}$ and ${\overset{˙˙}{y}}_{0}$ are the current longitudinal and lateral acceleration, respectively, $x_{f}$ and $y_{f}$ are the final longitudinal and lateral position, respectively, ${\dot{x}}_{f}$ and ${\dot{y}}_{f}$ are the final longitudinal and lateral velocity, respectively, ${\overset{..}{x}}_{f}$ and ${\overset{..}{y}}_{f}$ are the final longitudinal and lateral acceleration, respectively.

Then the state matrix of subject vehicle can be represented as $z_{x}$ and $z_{y}$:

(7) $$z_{x}=\left[\begin{array}{cccccc} x_{0} & {\dot{x}}_{0} & {\overset{˙˙}{x}}_{0} & x_{f} & {\dot{x}}_{f} & {\overset{˙˙}{x}}_{f} \end{array}\right]\;z_{y}=\left[\begin{array}{cccccc} y_{0} & {\dot{y}}_{0} & {\overset{˙˙}{y}}_{0} & y_{f} & {\dot{y}}_{f} & {\overset{˙˙}{y}}_{f} \end{array}\right]$$

In this study, the specific values of state matrix can be defined as follows:

(8) $$\left\{\begin{array}{c} \;\;z_{x}=\left[\begin{array}{cccccc} x_{0} & v_{SV} & a_{SV} & x_{f} & v_{des}^{l} & a_{SV}{}^{\prime } \end{array}\right]\\ z_{y}=\left[\begin{array}{cccccc} y_{0} & 0 & 0 & y_{f} & 0 & 0 \end{array}\right]\;\;\;\;\;\;\;\;\;\;\;\;\;\;\;\;\; \end{array}\right.$$

**Figure 3:**
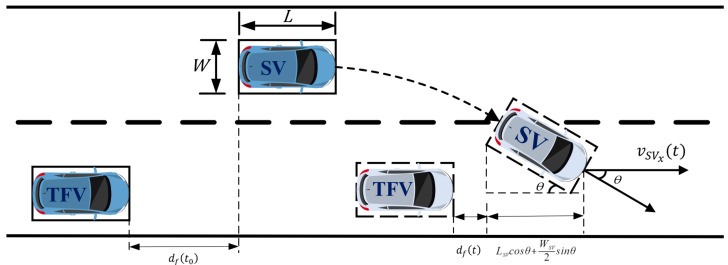
Insertable gab between *SV* and *TFV*.

In diverging area, to rule out the potential of a collision while changing lanes, the insertable gaps in TL should ensure that the *SV* always maintains safe distance from the vehicles ahead and behind during the lane change process. As shown in Figure 3, the longitudinal relative distance $d_{f}\left(t\right)$ between *SV* and target following vehicle (*TFV*) in the process of lane-changing should be satisfied as follows:

(9) $$d_{f}\left(t\right)=d_{f}\left(t_{0}\right)+∆d\left(t\right)+L_{SV}-L_{SV}cos\theta -\frac{W_{SV}}{2}sin\theta \geq s_{0}$$

where θ is the heading angle,$\;L_{SV}$ and $W_{SV}$ are the length and width of *SV*, respectively, $d_{f}\left(t_{0}\right)$ is relative distance between *SV* and *TFV* at starting of lane-changing while $∆d\left(t\right)$ is variation at time t, which are calculated as follows:

(10) $$d_{f}\left(t_{0}\right)=x_{SV}\left(t_{0}\right)-x_{TFV}\left(t_{0}\right)-L_{SV}$$

(11) $$∆d\left(t\right)=\left[v_{SV_{x}}\left(t_{0}\right)-v_{TFV}\left(t_{0}\right)\right]\cdot \left(t-t_{0}\right)+\overset{t}{\underset{t_{0}}{\int }}\overset{\lambda }{\underset{0}{\int }}\left[a_{SV_{x}}\left(\tau \right)-a_{TFV}\left(\tau \right)\right]d\tau d\lambda$$

where $x\left(t\right)$ is the longitudinal location at time t, $v\left(t\right)$ is the velocity at time t, $a\left(t\right)$ is the acceleration at time t; $v_{SV_{x}}\left(t\right)$ and $a_{SV_{x}}\left(t\right)$ are longitudinal velocity and acceleration of *SV* at time t, respectively.

**Figure 4:**
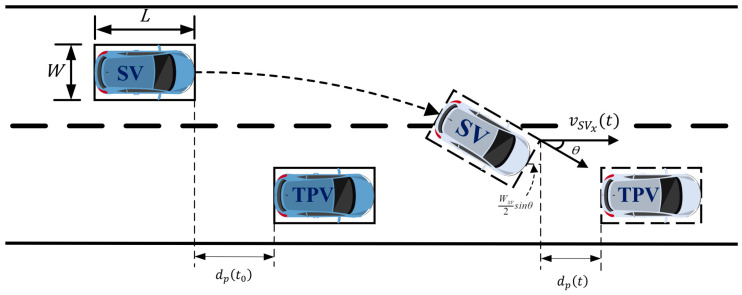
Insertable gab between *SV* and *TPV*.

Similarly, according to Figure 4, we can obtain $d_{p}\left(t\right)$, the relative distance between *SV* and target preceding vehicle (*TPV*) as follows:

(12) $$d_{p}\left(t\right)=d_{p}\left(t_{0}\right)+∆d\left(t\right)-\frac{W_{SV}}{2}sin\theta \geq s_{0}$$

where the relative distance between *SV* and *TPV* $d_{p}\left(t_{0}\right)$ and $∆d\left(t\right)$ can be calculated as follows:

(13) $$d_{p}\left(t_{0}\right)=x_{TPV}\left(t_{0}\right)-x_{SV}\left(t_{0}\right)-L_{TPV}$$

(14) $$∆d\left(t\right)=\left[v_{TPV}\left(t_{0}\right)-v_{SV_{x}}\left(t_{0}\right)\right]\cdot \left(t-t_{0}\right)+\overset{t}{\underset{t_{0}}{\int }}\overset{\lambda }{\underset{0}{\int }}\left[a_{TPV}\left(\tau \right)-a_{SV_{x}}\left(\tau \right)\right]d\tau d\lambda$$

As a consequence, it can be determined that the minimum insertable gap for the TL requires to comply with the following requirements:

(15) $$\left\{\begin{array}{c} d_{f_{min}}=\max\left\{s_{0}+L_{SV}cos\theta +\frac{W_{SV}}{2}sin\theta -∆d\left(t\right)-L_{SV}\right\},\;\;\;t\in \left[t_{0},\;t_{f}\right]\\ d_{p_{min}}=\max\left\{s_{0}+\frac{W_{SV}}{2}sin\theta -∆d\left(t\right)\right\},\;\;\;\;\;\;\;\;\;\;\;\;\;\;\;\;\;\;\;\;\;\;\;\;\;\;\;\;\;\;\;\;\;\;\;\;t\in \left[t_{0},\;t_{f}\right] \end{array}\right.$$

In the process of lane-changing, the collision avoidance function aforementioned can ensure the safety of *SV* movements. Nevertheless, the comfort of the driver will be jeopardized if the lateral acceleration is over threshold. Hence, the lateral acceleration $a_{l}$ should be lower than the threshold $a_{yc}$, which is the level of human-comfortable lateral acceleration. $a_{l}$ is calculated as follows:

(16) $$a_{l}=v^{2}\kappa =v^{2}\frac{\left|y^{\prime \prime }\left(x\right)\right|}{{\left[1+{y^{\prime }}^{2}\left(x\right)\right]}^{\frac{3}{2}}}=\frac{\left|y^{\prime \prime }\left(t\right)x^{\prime }\left(t\right)-x^{\prime \prime }\left(t\right)y^{\prime }\left(t\right)\right|}{{\left[{x^{\prime }}^{2}\left(t\right)+{y^{\prime }}^{2}\left(t\right)\right]}^{\frac{1}{2}}}$$

where κ is the curvature of vehicle trajectory.

### 2.3. Motion Prediction

For the purpose of formulating the most efficient control driving strategy in the whole-time horizon, *SV* should have the ability to perceive the state of vehicles in diverging influence area. However, the V2X technology can only provide the real-time motion state of vehicles in diverging area. Thus, a vehicle motion state prediction model considering communication time delay is proposed in this study. We define the prediction horizon is p, number of predictable vehicles n, prediction time step τ and vehicle position state x, which is longitudinal location. Then the position state variable of the vehicle can be written as:

(17) $$x\left(k\right)={\left[x_{1}\left(k\right),x_{2}\left(k\right),\ldots ,x_{n}\left(k\right)\right]}_{n\times 1}^{T}\;$$

The update forms of vehicle position state can be written as:

(18) $$x\left(k+\tau |k\right)=x\left(k\right)+\dot{x}\left(k\right)\tau +\frac{1}{2}\tau^{2}B\left(k\right)u\left(k\right)+C\left(k\right)a\left(k\right)\;$$

where $x\left(k\right)$ is actual measurement value while $x\left(k+\tau |k\right)$ is the prediction value based on $x\left(k\right)$; $\dot{x}\left(k\right)$ is the velocity vector, $B\left(k\right)$ is the control weighting factor matrix, which presents the extent of adoption of the car-following model by different vehicles at moment k; $C\left(k\right)$ is the error weighting factor matrix, which presents the state prediction error at moment k due to the communication time delay and system response delay;$\;a\left(k\right)$ is the actual accelerations shown in Equation (19), $B\left(k\right),C\left(k\right)$ are *n*-order diagonal matrixes shown expressed by Equation (20):

(19) $$a\left(k\right)={\left[{\ddot{x}}_{1}\left(k\right),{\ddot{x}}_{2}\left(k\right),\ldots ,{\ddot{x}}_{n}\left(k\right)\;\right]}_{n\times 1}^{T}\;$$

(20) $$\left\{\begin{array}{c} B\left(k\right)=diag\left[\gamma_{1}\left(k\right),\gamma_{2}\left(k\right),\ldots ,\gamma_{n}\left(k\right)\right]\;\;\;\;\;\;\\ C\left(k\right)=\frac{1}{2}diag{\left[\sigma_{1}\left(k\right),\sigma_{2}\left(k\right),\ldots ,\sigma_{n}\left(k\right)\right]}^{2} \end{array}\right.$$

Since HDVs and CAVs adopt different following models in longitudinal motion, and driving styles of different drivers also lead to the differences in the adoption of following models in actual traffic flow, we fully consider the differences of drivers when predicting the acceleration of surrounding vehicles.$\;\hat{u}\left(k\right)$ is the input prediction acceleration, which are denoted and calculated as follows:

(21) $$\hat{u}\left(k\right)={\left[{\hat{u}}_{1}\left(k\right),{\hat{u}}_{2}\left(k\right),\ldots ,\;{\hat{u}}_{n}\left(k\right)\;\right]}_{n\times 1}^{T}\;$$

(22) $${\hat{u}}_{i}\left(k\right)=\left\{\begin{array}{c} {\ddot{x}}_{i}\left(k\right)\;\;\;\;\;\;\;\;\;\;\;\;\;\;\;\;\;\;\;\;\;\;\;\;\;\;\;\;\;\;\;\;\;\;\;\;\;\;\;\;\;\;\;\;\;\;\;\;\;\;\;\;\;\;\;,\;\;\;i=n\;\;\;\;\;\;\;\;\;\;\;\;\;\;\;\;\;\;\;\;\;\\ \left[\alpha_{i}\left(x_{i}\left(k\right),{\dot{x}}_{i}\left(k\right),x_{i+1}\left(k\right),{\dot{x}}_{i+1}\left(k\right)\right)\right],\;\;\;i=1,2,\ldots ,n-1 \end{array}\right.$$

where $\alpha_{i}\left(\cdot \right)$ is the car-following model of vehicle i. The predicted acceleration values of the surrounding vehicles of *SV* can then be calculated as $B\left(k\right)\hat{u}\left(k\right)$, accounting for the variations in drivers.

Based on Equation (18)–(22), we can calculate the vehicle states at moment k+jτ:

(23) $$x\left(k+j\tau |k\right)=x\left(k+\left(j-1\right)\tau \right)+\dot{x}\left(k+\left(j-1\right)\tau \right)\tau \;\;\;\;\;\;\;\;\;\;\;\;\;\;\;+\frac{1}{2}\tau^{2}B\left(k\right)\hat{u}\left(k+\left(j-1\right)\tau |k\right)+C\left(k\right)a\left(k+\left(j-1\right)\tau \right)\;$$

Then the states at all times in the prediction time horizon can be obtained from the recursion relation on moment k, which is described as follows:

(24) $$x\left(k+j\tau |k\right)=x\left(k\right)+\tau \left[\dot{x}\left(k\right)+\dot{x}\left(k+1\right)+\ldots +\dot{x}\left(k+\left(j-1\right)\tau \right)\right]\;\;\;\;\;\;\;\;\;\;\;+\frac{1}{2}\tau^{2}B\left(k\right)\left[\hat{u}\left(k|k\right)+\hat{u}\left(k+\tau |k\right)\ldots +\hat{u}\left(k+\left(j-1\right)\tau |k\right)\right]\;\;\;\;\;\;\;\;\;\;\;\;\;\;+C\left(k\right)\left[a\left(k\right)+a\left(k+\tau \right)+\ldots +a\left(k+\left(j-1\right)\tau \right)\right],\;j=1,2,\ldots ,p\;$$

Once the prediction of position state was completed, the future motion state $s_{i}\left(j\right)$ in the prediction horizon p can be obtained:

(25) $$s_{i}\left(k+j\tau \right)={\left[x_{i}\left(k+j\tau \right),v_{i}\left(k+j\tau \right),a_{i}\left(k+j\tau \right)\right]}^{T},i=1,2,\ldots ,n,j=1,2,\ldots ,p\;$$

We define $S_{l}$ as flow state matrix of lane l, which is shown as follows:

(26) $$S_{l}={\left[\begin{array}{ccc} s_{1}\left(k\right) & \cdots & s_{1}\left(k+p\tau \right)\\ \vdots & \ddots & \vdots \\ s_{n_{l}}\left(k\right) & \cdots & s_{n_{l}}\left(k+p\tau \right) \end{array}\right]}_{n_{l}\times p},l\in \left[l_{1},\;l_{2},l_{3}\right]\;$$

### 2.4. Feasible Strategies Generation

According to flow state matrix $S_{l}$, $\left(n_{l}-1\right)\times p$ gaps can be calculated. We use $G_{ij}^{l}$ to denote the state of gap i at moment j in lane l, where i=1,2,…,n−1, j=1,2,…,p. The value of $G_{ij}^{l}$ is set as $G_{ij}=1$ if the following conditions are satisfied else $G_{ij}^{l}=0$:

(27) $$\left\{\begin{array}{c} x_{SV}\left(k+j\tau \right)-x_{TFV}^{i}\left(k+j\tau \right)-L_{SV}\geq d_{f_{min}}\left(k+j\tau \right)\;\;\;\\ x_{TPV}^{i}\left(k+j\tau \right)-x_{SV}\left(k+j\tau \right)-L_{TPV}\geq d_{p_{min}}\left(k+j\tau \right) \end{array}\right.\;$$

Then we can obtain the gap matrix $G^{l}$ made up of zeros and ones shown as follows:

(28) $$G^{l}\in {\mathbb{R}}_{\left(n_{l}-1\right)\times p},\;l\in \left[l_{1},\;l_{2},l_{3}\right]\;$$

We assume that the nearest lane of off-ramp is lane 3. Thus, the *SV* is supposed to arrive at $G_{ij}^{3}$ before diverging finish for the purpose of complete the driving task. Due to the limitation of spatial location, vehicles are only able to change to Adjacent lanes. This study defines the strategy of lane-changing when the vehicle locates on lane 1, lane 2 and lane 3, as shown:

(29) $$\left\{\begin{array}{c} f\left(G_{ij}^{l_{1}},G_{ij}^{l_{2}}\right)\;\;\;\;\;\;\;\;\;\;\;\;\;\;\;\;\;\;\;\;\;\;\\ f\left(G_{ij}^{l_{2}},G_{ij}^{l_{1}}\right),\;f\left(G_{ij}^{l_{2}},G_{ij}^{l_{3}}\right)\\ f\left(G_{ij}^{l_{3}},G_{ij}^{l_{2}}\right)\;\;\;\;\;\;\;\;\;\;\;\;\;\;\;\;\;\;\;\;\;\; \end{array}\right.\;$$

where $f\left(G_{ij}^{l_{1}},G_{ij}^{l_{2}}\right)$ denotes the strategy of lane-changing from $G_{ij}^{l_{1}}$ to $G_{ij}^{l_{2}}$ of *SV*. The strategy adopts the fifth-degree polynomial as lane-changing model shown in Equation (4) and includes all the acceleration, deceleration and lane-changing information of the *SV* from state $s_{G_{ij}^{l_{1}}}$ to state $s_{G_{ij}^{l_{2}}}$, shown as follows:


(30) $$f\left(G_{ij}^{l_{1}},G_{ij}^{l_{2}}\right)=\left[s_{G_{ij}^{l_{1}}}\overset{a\left(t\right),\;z_{x,}\;z_{y}}{\to }s_{G_{ij}^{l_{2}}}\right]\;$$

Based on lane-changing strategy, the whole strategy of driving through the diverging area could be formulated, which is denoted by $\pi \left(G_{ij}^{3}=1\right)$ shown as follows:

(31) $$\pi \left(G_{ij}^{l_{3}}=1\right)=F_{SV}\left[f_{1}\left(G_{0},G_{ij}^{l}\right),f_{2}\left(G_{ij}^{l},G_{ij}^{l}\right),\ldots ,f_{N-1}\left(G_{ij}^{l},G_{ij}^{l_{3}}\right),f_{N}\left(G_{ij}^{l_{3}},G_{ramp}\right)\right]\;$$

$$s.t.\;\;\;G_{ij}^{l}=1,\;l\in \left[l_{1},\;l_{2},l_{3}\right]$$

$$\left|a_{l}(t)\right|\leq a_{yc}\;\;\;\;\;\;\;\;\;\;\;\;\;\;\;\;\;\;\;$$

$$v_{min}\leq v_{f_{N}}\left(t\right)\leq v_{max}$$

$$a_{min}\leq a_{f_{N}}\left(t\right)\leq a_{max}$$

$$d\left(SV,PV\right)<v\left(t\right)T+s_{0}$$

$$d\left(SV,FV\right)<v\left(t\right)T+s_{0}$$

where *N* is lane-changing times;$\;a_{l}(t)$ is the lateral acceleration while $a_{yc}$ is the human-comfortable lateral acceleration;$\;v_{min},v_{max}$ are the minimum and maximum velocities of *SV*, respectively;$\;a_{min},a_{max}$ are the minimum and maximum accelerations of *SV*, respectively; $d\left(SV,PV\right)$ and $d\left(SV,FV\right)$ denote the relative distance of *SV* between *PV* and *FV*, respectively; $f\left(G_{ij}^{l},G_{ij}^{l}\right)$ complies with Equation (29).

All feasible strategies for the diverging area are calculated denoted by Ρ:

(32) $$Ρ=\left[\pi_{1},\pi_{2},\ldots ,\pi_{w}\right]\;$$

### 2.5. Performance Measurement

The constraints in the previously suggested technique can effectively guarantee the safety and comfort of the vehicle-driving experience. The purpose of this study, however, is to suggest a driving strategy with optimal efficiency in the full-time horizon; as a result, the efficiency of the proposed driving strategy must be fully considered. Nevertheless, as road resources are limited, *SV* cannot concentrate exclusively on its own efficiency; otherwise, it would have an effect on the overall traffic flow in the diverging area. An evaluation function that considers both *SV* efficiency and the overall operational efficiency of the traffic flow in the diverging area is defined. $J_{SV}$ is the *SV* efficiency shown as:

(33) $$J_{SV}=\frac{X\left(G_{0},G_{ramp}\right)}{v_{des}\cdot \sum_{h=1}^{N}T\left(f_{h}\right)}\;$$

where $X\left(G_{0},G_{ramp}\right)$ is the distance between the current position $G_{0}$ and the arrival position of off-ramp $G_{ramp}$, $T\left(f_{h}\right)$ is the duration of lane-changing strategy $f_{h}$.

$J_{flow}$ is the overall operational efficiency of the traffic flow in the diverging area shown as:

(34) $$J_{flow}=\frac{1}{n}\overset{3}{\underset{l=1}{\sum }}\overset{n_{l}}{\underset{i}{\sum }}\frac{{\bar{v}}_{i}^{l}}{v_{des}}\;$$

where n is the total number of vehicles in diverging area, calculated by $n=n_{1}+n_{2}+n_{3}$; ${\bar{v}}_{i}^{l}$ represents the average velocity of vehicle i in lane l.

Time to Collision (*TTC*) is time-based and considers both spatial proximity and speed differential [48]. It is used as a threat metric to assess the probability of a collision between *SV* and another vehicle. The formulas for *TTC* is set as follows:

(35) $$TTC_{i}=\frac{x_{i-1}\left(t\right)-x_{i}\left(t\right)-L}{v_{i}\left(t\right)-v_{i-1}\left(t\right)}\;$$

Then the safety evaluation function for *SV* is defined as follows:

(36) $$J_{TTC}=\frac{1}{4}\sum \frac{TTC_{j}}{\bar{TTC}},\;j\in \left(SV,SV_{TL},FV,TFV\right)\;$$

where $TTC_{SV}$ presents the *TTC* between *SV* and *PV*, $TTC_{SV_{TL}}$ presents the *TTC* between *SV* and *TPV*, $TTC_{FV}$ presents the *TTC* between *FV* and *SV*, $TTC_{TFV}$ presents the *TTC* between *TFV* and *SV*.

Eventually, the evaluation function is:

(37) $$J\;=\;\xi_{1}J_{SV}\;+\;\xi_{2}J_{flow}+\xi_{3}J_{TTC}\;$$

where $\xi_{1}$, $\xi_{3}$ and $\xi_{2}$ are weighting factors, complied with $\xi_{1}+\xi_{2}+\xi_{3}=1$.

### 2.6. Optimal Strategy Execution

For the propose of obtaining the optimal driving strategy in the full-time horizon, the MPC system apply the moving horizon optimization algorithm, which can be shown as follows:

(1) Based on the evaluation function and feasible strategies, the optimal strategy $\pi_{opt}$ could be calculated:

(38) $$\pi_{opt}=arg_{\pi \in P}\max J\left(\pi \right)\;$$

(2) In the next mτ time horizons, the *SV* execute the optimal lane change strategy $\pi_{opt}$. Once the execution is completed, the prediction error in this time horizons could be calculated, which is shown as follows:

(39) $$e_{i}=\frac{\left|x(k+j\tau )-x(k+j\tau |k)\right|}{x(k+j\tau )}\;$$

(40) $$e={\left[\begin{array}{ccc} e_{11} & \cdots & e_{1m}\\ \vdots & \ddots & \vdots \\ e_{n1} & \cdots & e_{nm} \end{array}\right]}_{n\times m}$$

where $e_{i}$ is the prediction error of vehicle i and e is the error matrix.

Then elements of $B\left(k\right)$ and $C\left(k\right)$ can be updated as follows:

(41) $$\gamma_{i}\left(k+j\tau \right)=\frac{1}{j}\left[\gamma_{i}\left(k+\left(j-1\right)\tau \right)\cdot \left(j-1\right)+\frac{u_{i}\left(k+\left(j-1\right)\tau \right)}{{\hat{u}}_{i}\left(k+\left(j-1\right)\tau |k+\left(j-2\right)\tau \right)}\right]\;$$

(42) $$\sigma_{i}\left(k\right)=arg_{\sigma_{i}}\min e_{i}$$

(3) The MPC system is supposed to execute the optimal strategy $\pi_{opt}$ if the prediction error e satisfy the following conditions:

(43) $$e_{ij}\leq \eta ,\;e_{ij}\in e\;$$

Otherwise, the MPC system will re-predict the vehicle’s motion state that cannot comply with the aforementioned constraints based on the current measurement values. Meanwhile, the feasible strategy matrix Ρ is updated, and the MPC system could re-formulate and execute the optimal strategy $\pi_{opt}{}^{\prime }$.

The MPC system would repeat steps (1–3) until the *SV* successfully changes lanes to the off-ramp in diverging area.

## 3. Experiment Design

**Figure 5:**
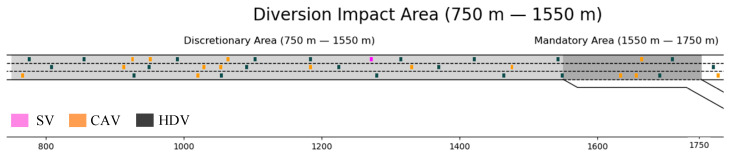
Traffic simulation environment.

In order to validate the effectiveness the proposed strategy, a case study was designed. The case study utilized traffic simulation data. Firstly, we built a simulation environment with Python programming language, as shown in Figure 5. In this environment, the DIA was set from 750 m to 1750 m with a total length of 1000 m, the DLA was set from 750 m to 1550 m, and the MLA was set from 1550 m to 1750 m. In the case study, we set the mainline freeway into three lanes and take the center line of the second lane as the *x*-axis, with the right direction as the traffic direction.

The freeway mainline has a three-lane total traffic flow of 4500 vehicles per hour, with HDVs distributed at random along each lane according to the Poisson distribution. The length of the vehicles is 4.3 m while the width is 1.8 m. In each complete experiment, the simulation time is 150 s, and the time step is 0.1 s. Assuming that the CAVs platoon degenerates to CAVs driving alone, then only the IDM model for human-driven vehicles and the ACC model for CAVs need to be taken into consideration. The following lists particular parameters:

For the IDM model, the desired acceleration and deceleration are 5 $m/s^{2}$ and −5 $m/s^{2}$, respectively. The safe headway is 1.8 s. The minimum distance at a standstill is 5 m.

For ACC and CACC models, the minimum distance at a standstill is 5 m. The comfortable acceleration is 3 $m/s^{2}$. The max speed is 33.3 m/s. The max acceleration and deceleration are 5 $m/s^{2}$ and −8 $m/s^{2}$, respectively. The system response time is 0.1 s. According to the calibration based on the field testing by Milanes and Shladover [15], the parameters of ACC models are set as $t_{a}=1.1\;s$, $k_{1}=0.23$ and $k_{2}=0.07$, while the parameters of the CACC model are set as $t_{c}=0.6\;s$, $k_{1}=0.45$ and $k_{3}=0.25$. The case took the average lane-changing time of 3 s of actual freeway data as the time of lane change model. The human-comfortable lateral acceleration threshold $a_{yc}$ was set as 3 $m/s^{2}$. As to the weighting factors of the evaluation function, the value of $\xi_{3}$ is set to the minimum since security constraints have been added to the calculation of the insertable gap. The proposed strategy is to improve the driving efficiency of a single CAV on the premise of not affecting the operating efficiency of overall traffic flow. Therefore, the coefficient of driving efficiency of single CAV $\xi_{1}$ is set to the maximum while $\xi_{2}$ is the second largest. The weighting factors of the evaluation function are set as $\xi_{1}=0.5$, $\xi_{2}=0.3\;$ and $\xi_{3}=0.2$.

In order to confirm the validity of the proposed model, we designed two typical driving styles in real-world circumstances as control tests, dubbed “ Conservative driver” and “aggressive driver”. The “Conservative driver” means that the vehicle instantly begins to search for the insertable gap when it enters the DIA. If there is no insertable gap, it will slow down properly until it finds the insertable gap and changes lanes. This driving style is more likely to move into the lane closest to the off-ramp in DLA. The “Aggressive driver” refers to the vehicle tending to pursue higher driving efficiency; thus, it will not start to find the insertion gap and change lanes in the DLA until the vehicle enters the MLA.

In the initial network, after the arterial traffic flow is generated according to Poisson distribution, the time of traffic flow generation and random initial speed are recorded to form the arterial dataset. The experiment is divided into three steps:

Step 1. At the beginning of the experimental simulation, a dataset with 50% of CAVs and 50% of HDVs is loaded to simulate the traffic operation in normal mode in the diverging area. As traffic flow stabilized, the *SV* was generated at the 32 s time in $L_{1}$. Eight sets of control experiments were conducted to verify the effectiveness of the proposed strategy. The first was the “Conservative driver” lane-changing experiment, which was a random lane change after the *SV* entered the DLA. Then a seven-group random lane-changing experiment was conducted for *SV* to start to change lanes in DLA. Finally, a set of “Aggressive driver” lane change trials were conducted after the *SV* entered the MLA. Meanwhile, the safety and comfort constraints proposed in this paper are always satisfied during the lane change and the following driving. The velocity, acceleration, and position data of all vehicles in the diverging area (mainline and diverging traffic) were recorded after the experiments.

Step 2. Reload the dataset with 50% of CAVs and 50% of HDVs to simulate the traffic operation in normal mode in the diverging area. Load the *SV* at the same time and position. Another set of experiments was applied to the strategy presented in this paper. The prediction model and MPC system were applied to generate the optimal strategy. In the same way, the speed, acceleration, and position data of all vehicles at the whole time in the diverging area were recorded as “Proposed strategy”. Compare the difference between “Conservative driver” and “Proposed strategy” to analyze the superiority of the proposed model.

Step 3. Initialize the traffic flow environment with the different datasets consisting of different proportions of CAVs and HDVs, and repeat the aforementioned Step 1 and 2. In the same way, the speed, acceleration, and position data of all vehicles at the whole time in the diverging area were recorded as more persuasive experiments.

## 4. Result Analysis

Based on the datasets obtained from the above experiments, the real-time position of *SV*, velocity of *SV*, longitudinal and lateral acceleration of *SV*, the distance between *SV* and surrounding vehicles, lane-changing duration, and diverging traffic flow operation was assessed to investigate the validation of the proposed strategy.

**Figure 6:**
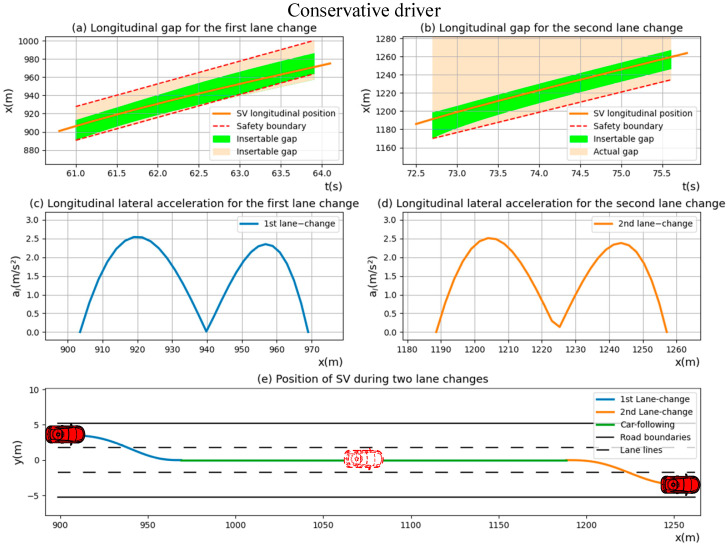
The position, lateral acceleration and trajectories of “Conservative driver” during two lane-changing processes.

Firstly, a group of “Conservative driver” datasets were randomly selected to visualize the lane change driving strategy, as shown in Figure 6. It can be seen from Figure 6a,b that the relative distance between *SV* and the vehicles ahead and behind the *SV* is exactly sufficient to meet the insertable gap requirement during the first lane-changing process. The actual relative distance between *SV* and *TPV* is significantly greater than the insertable gap, leading to the fact that *SV* has no need to consider the influence of the vehicle in front of it during the lane-changing process. These results show that *SV* is capable of maintaining safety at all times during the lane-changing process.

Combining the analysis in Figure 6c–e, it can be found that the lateral acceleration of the lane-changing trajectories of *SV* is always within the comfort level of the driver, and the lateral acceleration of each completely lane-changing process would reach two peaks, which are one quarter and three-quarters of the lane-changing trajectory respectively, indicating that the comfort of the whole lane-changing process can be ensured by keeping the acceleration at this point less than the human-comfortable acceleration threshold.

**Figure 7:**
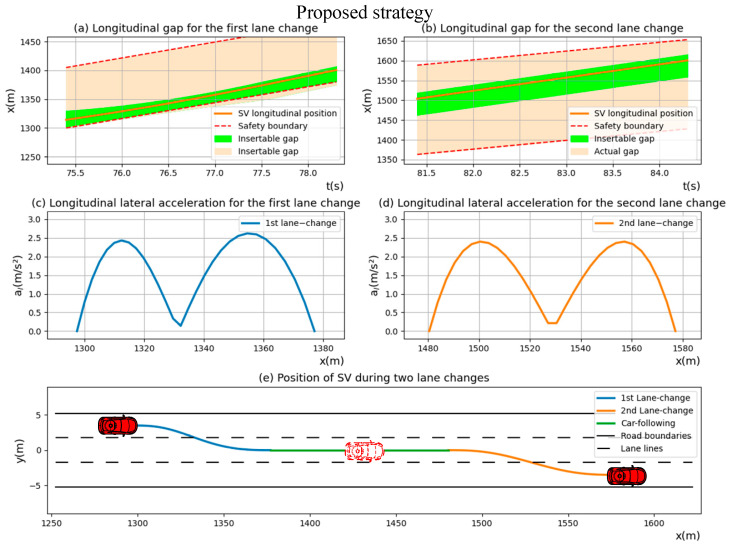
The position, lateral acceleration and trajectories of “Proposed strategy” during two lane-changing processes.

Simultaneously, the proposed strategy of lane-changing in diverging areas for *SV* was visualized in Figure 7. Compared with the simulation results of “Conservative driver”, the proposed strategy can not only ensure safety and comfort during lane-changing but also make the car-following distance between two lane changes of *SV* shorter, which dramatically improves the lane-changing efficiency.

**Figure 8:**
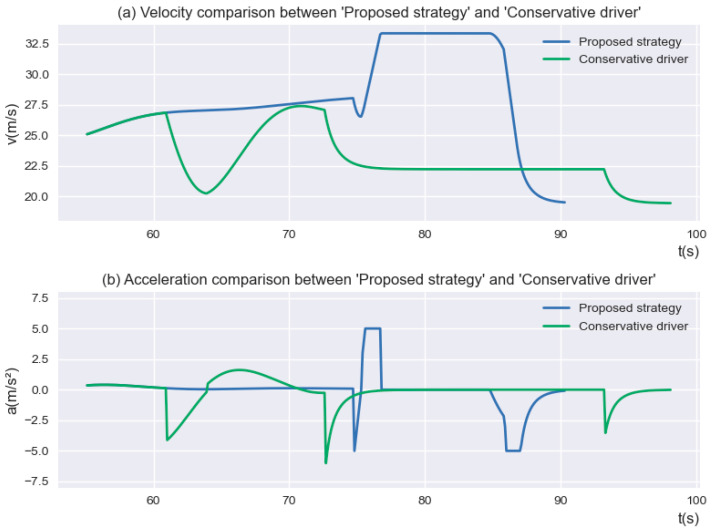
The velocity and acceleration of “Proposed strategy” and “Conservative driver”.

After that, the comparison of the velocity and acceleration between the “Conservative driver” and the proposed strategy is shown in Figure 8. It is apparent from Figure 8 that the velocity of the *SV* according to the proposed strategy is almost always higher than that of the “Conservative driver”.

Moreover, numerous simulation experiments are performed to verify the generalization performance of the proposed strategy based on the proportion of different CAVs. It can be seen from the results of Table 1 that as the proportion of CAV increases, the difference between the proposed driving strategy and the “conservative driver” in terms of speed and travel time becomes greater. That is to say, the higher the proportion of CAV in the traffic flow, the more significant the efficiency improvement of the proposed strategy on *SV* itself. Notably, the average travel time and velocity of each lane did not change significantly with this significant increase, which means that the *SV* self-efficiency does not result in a decrease in overall traffic flow efficiency.

**Figure 9:**
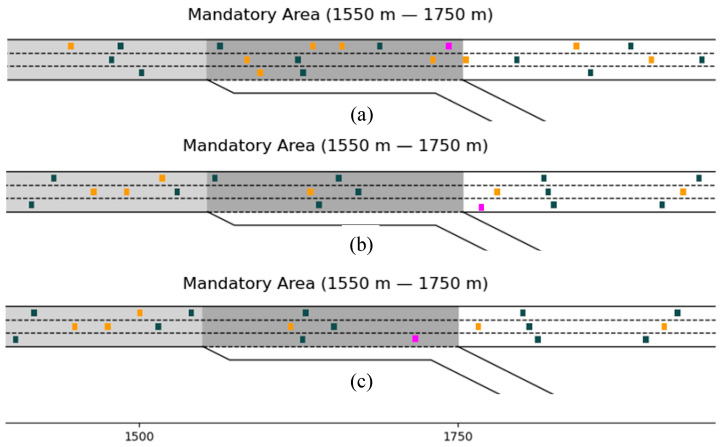
“Aggressive Driver” lane-changing simulation scenario. (**a**) and (**b**) are the “Aggressive Drivers” who failed to drive away from the diverging area, and (**c**) is the “Aggressive Driver” who succeeded.

Both the “Conservative driver” and the “Proposed strategy” enable the *SV* to make a successful lane-changing maneuver from the $L_{1}$ to the off-ramp so that the travel task could be completed. However, as for the “Aggressive driver”, as shown in Figure 9, although the *SV* made a considerable reduction in braking to seek the insertable gap after entering the MLA, the *SV* in the experiment still missed the diverging opportunity by the first time it completed the lane-changing maneuver due to the large volume of traffic and the high velocity of the vehicles in $L_{1}$ and $L_{2}$. Thus the *SV* failed to complete the travel off the ramp.

**Figure 10:**
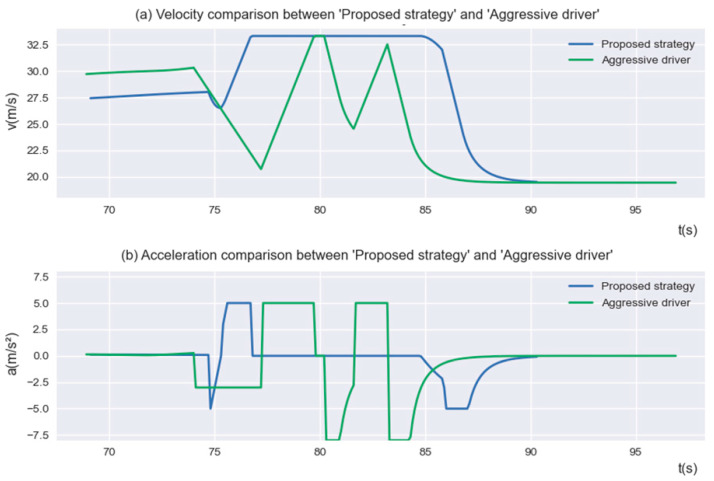
The velocity and acceleration of “Proposed strategy” and “Aggressive driver”.

To successfully change lanes and exit, the “Aggressive Driver” needs to slow down for a long time to find the insertable gap. We selected a group of “Aggressive Driver” successful lane change experiments to compare the velocity and acceleration of the proposed strategy, as shown in Figure 10. It can be seen from the figure that, in order to search for a suitable insertable gap in the MLA, the “Aggressive Driver” frequently accelerated and decelerated sharply, the velocity showed great fluctuation, and the average driving velocity was significantly lower than the proposed strategy.

**Figure 11:**
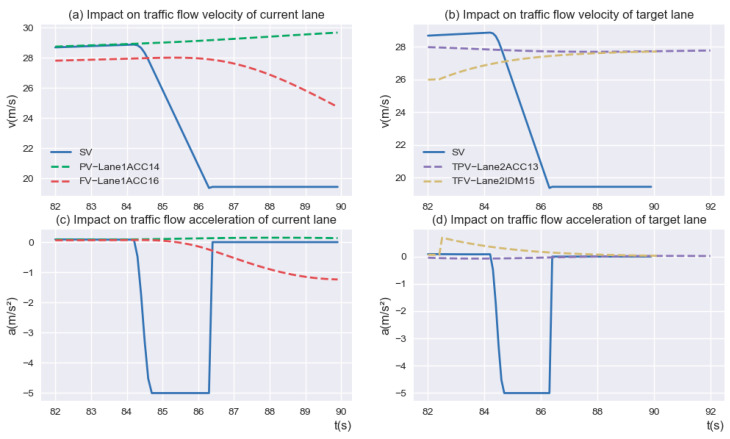
Impact on ahead and behind vehicles of “Aggressive driver” driving.

Interestingly, as can be seen from the curves in Figure 11, the radical deceleration of the “Aggressive driver” after entering the MLA has little impact on the vehicle ahead (i.e., “Lane1ACC16”) and vehicles in the target lane (i.e., “Lane2ACC13” and “Lane2IDM15”). In contrast, it has a significant impact on the vehicle behind the current lane. The *SV* decelerates aggressively after 84 s to seek the insertable gaps, and the velocity and acceleration of the vehicle behind the current lane (i.e., “Lane1ACC14”) fluctuate dramatically, which may lead to the loss of string stability for the following traffic.

**Figure 12:**
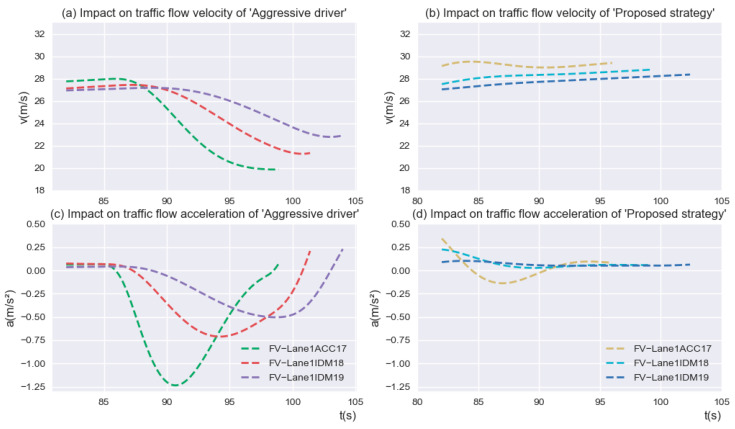
Impact on traffic flow operation of “Aggressive driver” and “Proposed strategy”.

To further investigate the impact of “Aggressive drivers” on the traffic flow behind the current position of *SV*, the speed acceleration variation of the second to fourth vehicles behind the *SV* was analyzed, as shown in Figure 12. The motion state of the corresponding vehicles without lane-changing maneuvers was used as a comparison. The results showed that although the rear traffic flow exhibited the characteristics of string stability, compared with the traffic flow operation under the “Proposed strategy”, the rear traffic flow showed great volatility and an overall decrease in driving velocity, which greatly affected the operational efficiency of the traffic flow in the diverging area.

**Figure 13:**
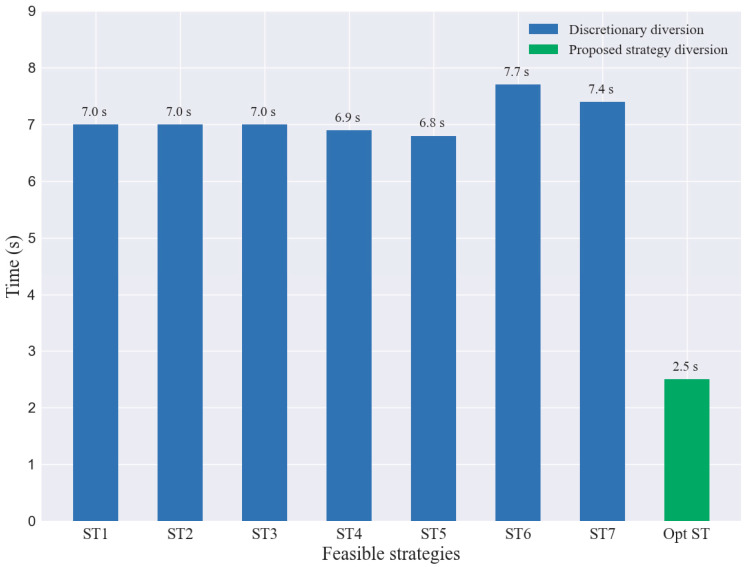
Comparison of loss time of feasible strategies.

On the contrary, the proposed strategy can predict and avoid the unsuccessful lane change dilemma encountered by “Aggressive drivers” in advance and execute the optimal strategy among the generated feasible strategies. In Table 2, seven sets of randomized experiments (ST1–ST7) and the travel time of SVs with the proposed strategy (Opt ST) in DIA are shown, and the results show that the proposed strategy can significantly improve the single-vehicle travel efficiency of SVs. Assuming that the desired speed of vehicle passage is 100 km/h and the desired travel time is 36 s, then the lost time of travel through the DIA for each strategy can be obtained, as shown in Figure 13. From the figure, the average travel loss time of the random lane change strategy is about 7 s, and the travel time of the optimal strategy is 2.5 s. The *SV* can reduce the loss time of traveling by more than 60% by following the proposed strategy.

**Figure 14:**
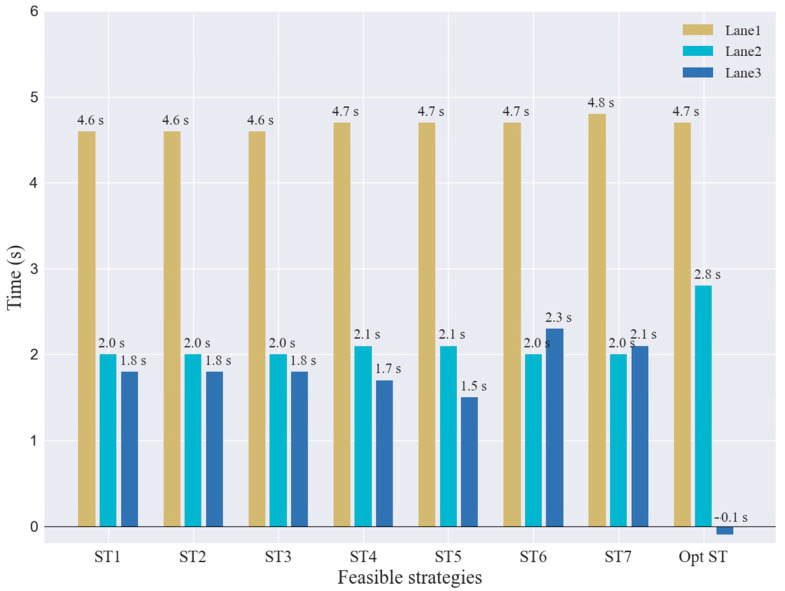
Impact on traffic flow operation of feasible strategies.

Moreover, the proposed strategy is also interested in the efficiency of the overall traffic flow. The desired travel velocity and desired travel time for each lane are defined as shown in Table 3. Figure 14 shows the overall operation of the traffic flow in the diverging area during the *SV* lane-changing, including the average loss time of traveling through each lane of the feasible strategy. Comparing the optimal and feasible strategies, the results show that the *SV* lane-changing maneuver does not affect the normal operation of the traffic flow for lane one. For lane two, the average loss time of vehicle traveling increases by 0.8 s, which is within the acceptable range. For lane three, the average loss time of surrounding flow traveling is significantly reduced, which indicates that the traveling of surrounding traffic flow is almost unaffected by the *SV* lane change driving.

## 5. Conclusions

This study proposed a lane change driving strategy for CAV in the diverging area using an MPC system. A vehicle kinematic model was first established, and the microscopic simulation of the vehicle lane change driving was carried out. Then a parameter-based vehicle motion prediction model was designed, and an MPC system was constructed to generate the optimal driving strategy. After that, Numerous simulation experiments were conducted to verify the effectiveness of the proposed strategy.

Based on the above traffic simulation experiments and discussion, the following two conclusions can be established:

(1) For CAV self-driving, the proposed strategy can not only ensure the safety and comfort of the CAV during the lane change driving process but also promote the efficiency of the CAV exiting to off-ramp significantly, resulting in over 60% reduction in lost time of traveling.
(2) For comprehensive traffic flow operation, the proposed driving strategy maintains the string stability of the car-following vehicles behind the CAV and also enhances the overall operating efficiency compared to the conventional lane change strategy.
(3) The proposed strategy is more efficient with a higher proportion of CAVs in the traffic flow, leading to less impact on the operational efficiency of the global traffic flow.

However, this study still has certain limitations, so the following future research directions can be taken into consideration:

(1) This study assumes that other vehicles in the diverging area do not have lane-changing behaviors, so predictions of the motivation of surrounding vehicles to change lanes need to be added inside the prediction module.
(2) The proposed strategy does not consider the assistance of surrounding CAVs, and future research could concentrate on the investigation of cooperative lane change driving strategy.
(3) The objective of this paper is to examine individual CAV driving strategies, and subsequent research can be conducted to manage how the CAV platoon implements an efficient lane-changing out of the diverging area.

## Figures and Tables

**Table 1:** The proposed strategy performance in different CAVs proportion.

Percentage of CAV	Driving Style	Average Value	Lane 1	Lane 2	Lane 3	*SV*
CAV 0%	Conservative driver	Time (s)	39.6	43.6	48.3	41.0
		Velocity (m/s)	25.3	22.9	20.7	21.1
	Proposed Strategy	Time (s)	40.6	44.9	47.3	37.9
		Velocity(m/s)	24.6	22.3	21.1	22.8
CAV 30%	Conservative driver	Time (s)	31.3	39.5	45.8	41.0
		Velocity (m/s)	32.0	25.3	21.8	21.5
	Proposed Strategy	Time (s)	31.2	38.0	44.9	30.4
		Velocity (m/s)	32.0	26.4	22.3	30.5
CAV 50%	Conservative driver	Time (s)	31.2	36.0	44.8	39.6
		Velocity (m/s)	32.1	27.8	22.3	21.8
	Proposed Strategy	Time (s)	31.3	35.9	45.0	31.0
		Velocity (m/s)	31.9	27.8	22.2	27.9
CAV 70%	Conservative driver	Time (s)	31.0	37.1	44.3	41.0
		Velocity (m/s)	32.3	27.0	22.6	21.1
	Proposed Strategy	Time (s)	31.0	37.6	44.9	33.8
		Velocity (m/s)	32.2	26.6	22.3	29.4
CAV 100%	Conservative driver	Time (s)	30.9	35.4	44.9	41.0
		Velocity (m/s)	32.4	28.2	22.3	21.0
	Proposed Strategy	Time (s)	31.1	35.8	44.9	29.2
		Velocity (m/s)	32.2	27.9	22.3	31.3

**Table 2:** Travel time through the DIA of different strategies for *SV*.

Driving Strategy	ST1	ST2	ST3	ST4	ST5	ST6	ST7	Opt ST ^1^
Travel time(s)	43.0	43.0	43.0	42.9	42.8	43.7	43.4	38.5
Average velocity(m/s)	23.2	23.2	23.2	23.2	23.3	22.8	23	28.5
Maximum velocity(m/s)	27.4	27.4	27.4	28	28.8	27.2	27.3	33.3
Average acceleration ($m/s^{2}$)	−0.1	−0.1	−0.1	−0.1	−0.1	−0.1	−0.1	0.3
Maximum acceleration ($m/s^{2}$)	1.6	1.6	1.6	2	2.3	3	3.3	5
^2^ Std of velocity	2.3	2.3	2.3	2.5	2.7	2.5	2.8	2.9
^2^ Std of acceleration	1	1	1	1.2	1.4	1.1	1.2	1.7

^1^ Opt ST refers to the optimal driving strategy. ^2^ Std refers to standard deviation.

**Table 3:** Desired velocity and travel time for each vehicle travel through DIA.

	L1	L2	L3
Desired velocity (km/h)	110	100.0	80.0
Desired travel time (s)	31.3	36.0	45.0

## Data Availability

The data used to support the findings of this study are included within the article.

## Appendix A

**Table A1:** Nomenclature.

Symbol	Description
$PV_{n},\;FV_{n}$	The nth preceding and nth following vehicle of the current lane respectively
$TPV_{n},TFV_{n}$	The nth preceding and nth following vehicle of the target lane respectively
$a_{m}$	The maximum acceleration
$v_{d}$	The desired velocity
δ	The free acceleration exponent
$s^{*},s$	The expected and actual distance from the preceding vehicle respectively
$s_{0}$	The static safe distance
T	The desired time headway
b	The desired deceleration
∆v	The velocity difference from the preceding vehicle
∆x	The space headway
e	The space headway error
$t_{a},t_{c}$	The time headway of ACC and CACC model
$k_{1},k_{2},k_{3}$	The control parameters of ACC and CACC model
$v_{p}$	The velocity of the subject vehicle at the previous time step
$\dot{e}$	The derivative form of space headway error
$x\left(t\right),y\left(t\right)$	The longitudinal distance and lateral distance at time t respectively
$c_{0},\ldots ,c_{5},d_{0},\ldots ,d_{5}$	The coefficients parameters of fifth-degree polynomial model
$x_{0},{\dot{x}}_{0},{\overset{˙˙}{x}}_{0}$	The current longitudinal position, velocity and acceleration
$y_{0},{\dot{y}}_{0},{\overset{˙˙}{y}}_{0}$	The current lateral position, velocity and acceleration
$x_{f},{\dot{x}}_{f},{\overset{..}{x}}_{f}$	The final longitudinal position, velocity and acceleration
$y_{f},{\dot{y}}_{f},{\overset{..}{y}}_{f}$	The final lateral position, velocity and acceleration
θ	The heading angle
$L_{SV},W_{SV}$	The length and width of *SV*
$d_{f}\left(t_{0}\right)$	The relative distance between *SV* and *TFV* at starting of lane-changing
$d_{p}\left(t_{0}\right)$	The relative distance between *SV* and *TPV* at starting of lane-changing
$∆d\left(t\right)$	The variation at time t
$v_{SV_{x}}\left(t\right)$	The longitudinal velocity of *SV* at time t
$a_{SV_{x}}\left(t\right)$	The longitudinal acceleration of *SV* at time t
$a_{l}$	The transverse acceleration
$a_{yc}$	The level of human-comfortable lateral acceleration
κ	The curvature of *SV* trajectory
p,m	The prediction and control horizon of MPC system
n	The number of predictable vehicles
τ	The time step
$x\left(k\right)$	The actual measurement value of longitudinal position
$x\left(k+\tau |k\right)$	The prediction value based on $x\left(k\right)$
$B\left(k\right),C\left(k\right)$	The control and error weighting factor matrix
$a\left(k\right)$	The actual acceleration at moment k
$\hat{u}\left(k\right)$	The input prediction acceleration at moment k
$\alpha_{i}\left(\cdot \right)$	The car-following model of vehicle i
$S_{l}$	The flow state matrix of lane l
$f\left(G_{ij}^{l_{1}},G_{ij}^{l_{2}}\right)$	the strategy of lane-changing from $G_{ij}^{l_{1}}$ to $G_{ij}^{l_{2}}$ of *SV*
*N*	The lane-changing times
$a_{l}\left(t\right)$	The lateral acceleration
$d\left(SV,PV\right)$	The relative distance of *SV* between *PV*
$d\left(SV,FV\right)$	The relative distance of *SV* between *FV*
$X\left(G_{0},G_{ramp}\right)$	The distance between position $G_{0}$ and the arrival position of off-ramp $G_{ramp}$
$T\left(f_{h}\right)$	The duration of lane-changing strategy $f_{h}$
${\bar{v}}_{i}^{l}$	The average velocity of vehicle i in lane l
$TTC_{SV}$	The *TTC* between *SV* and *PV*
$TTC_{SV_{TL}}$	The *TTC* between *SV* and *TPV*
$TTC_{FV}$	The *TTC* between *FV* and *SV*
$TTC_{TFV}$	The *TTC* between *TFV* and *SV*
$\xi_{i}$	The weighting factors
$e_{i}$	The prediction error of vehicle i
e	The error matrix
$\pi_{opt}$	The optimal strategy developed by MPC system
$v_{min},v_{max}$	The minimum and maximum velocities limited by road conditions
$a_{min},a_{max}$	The minimum and maximum accelerations limited by vehicle self-limitation

## References

[1] Gao K., Yan D., Yang F., Xie J., Liu L., Du R., Xiong N.J.S. (2019). Conditional artificial potential field-based autonomous vehicle safety control with interference of lane changing in mixed traffic scenario. Sensors.

[2] Ahn S., Laval J., Cassidy M.J. (2010). Effects of merging and diverging on freeway traffic oscillations: Theory and observation. Transp. Res. Rec..

[3] Chen D., Ahn S. (2018). Capacity-drop at extended bottlenecks: Merge, diverge, and weave. Transp. Res. Part B Methodol..

[4] van Beinum A., Farah H., Wegman F., Hoogendoorn S. (2018). Driving behaviour at motorway ramps and weaving segments based on empirical trajectory data. Transp. Res. Part C Emerg. Technol..

[5] Zheng Z., Ahn S., Chen D., Laval J. (2011). Freeway traffic oscillations: Microscopic analysis of formations and propagations using wavelet transform. Procedia-Soc. Behav. Sci..

[6] Han Y., Ahn S. (2018). Stochastic modeling of breakdown at freeway merge bottleneck and traffic control method using connected automated vehicle. Transp. Res. Part B Methodol..

[7] Vegamoor V.K., Darbha S., Rajagopal K.R. (2019). A review of automatic vehicle following systems. J. Indian Inst. Sci..

[8] Bevly D., Cao X., Gordon M., Ozbilgin G., Kari D., Nelson B., Woodruff J., Barth M., Murray C., Kurt A. (2016). Lane change and merge maneuvers for connected and automated vehicles: A survey. IEEE Trans. Intell. Veh..

[9] Pan T., Guo R., Lam W.H., Zhong R., Wang W., He B. (2021). Integrated optimal control strategies for freeway traffic mixed with connected automated vehicles: A model-based reinforcement learning approach. Transp. Res. Part C Emerg. Technol..

[10] Dong C., Wang H., Li Y., Wang W., Zhang Z. (2019). Route control strategies for autonomous vehicles exiting to off-ramps. IEEE Trans. Intell. Transp. Syst..

[11] Li S., Li K., Rajamani R., Wang J. (2010). Model predictive multi-objective vehicular adaptive cruise control. IEEE Trans. Control Syst. Technol..

[12] Xiao L., Gao F. (2011). Practical string stability of platoon of adaptive cruise control vehicles. IEEE Trans. Intell. Transp. Syst..

[13] VanderWerf J., Shladover S., Kourjanskaia N., Miller M., Krishnan H. (2001). Modeling effects of driver control assistance systems on traffic. Transp. Res. Rec..

[14] Shladover S.E., Su D., Lu X.-Y. (2012). Impacts of cooperative adaptive cruise control on freeway traffic flow. Transp. Res. Rec..

[15] Milanés V., Shladover S.E. (2014). Modeling cooperative and autonomous adaptive cruise control dynamic responses using experimental data. Transp. Res. Part C Emerg. Technol..

[16] Gipps P.G. (1986). A model for the structure of lane-changing decisions. Transp. Res. Part B Methodol..

[17] Wang C., Sun Q., Li Z., Zhang H. (2020). Human-like lane change decision model for autonomous vehicles that considers the risk perception of drivers in mixed traffic. Sensors.

[18] Alizadeh A., Moghadam M., Bicer Y., Ure N.K., Yavas U., Kurtulus C. Automated lane change decision making using deep reinforcement learning in dynamic and uncertain highway environment. Proceedings of the 2019 IEEE intelligent transportation systems conference (ITSC).

[19] Chen S., Dong J., Ha P., Li Y., Labi S. (2021). Graph neural network and reinforcement learning for multi-agent cooperative control of connected autonomous vehicles. Comput.-Aided Civ. Infrastruct. Eng..

[20] Shi T., Wang P., Cheng X., Chan C.-Y., Huang D. Driving decision and control for automated lane change behavior based on deep reinforcement learning. Proceedings of the 2019 IEEE intelligent transportation systems conference (ITSC).

[21] Wang G., Hu J., Li Z., Li L. (2021). Harmonious lane changing via deep reinforcement learning. IEEE Trans. Intell. Transp. Syst..

[22] Dong J., Chen S., Li Y., Du R., Steinfeld A., Labi S. (2021). Space-weighted information fusion using deep reinforcement learning: The context of tactical control of lane-changing autonomous vehicles and connectivity range assessment. Transp. Res. Part C Emerg. Technol..

[23] Luo Y., Xiang Y., Cao K., Li K. (2016). A dynamic automated lane change maneuver based on vehicle-to-vehicle communication. Transp. Res. Part C Emerg. Technol..

[24] Suh J., Chae H., Yi K. (2018). Stochastic model-predictive control for lane change decision of automated driving vehicles. IEEE Trans. Veh. Technol..

[25] Xing Y., Lv C., Wang H., Wang H., Ai Y., Cao D., Velenis E., Wang F.-Y. (2019). Driver lane change intention inference for intelligent vehicles: Framework, survey, and challenges. IEEE Trans. Veh. Technol..

[26] Zhang Y., Ioannou P.A. (2016). Combined variable speed limit and lane change control for highway traffic. IEEE Trans. Intell. Transp. Syst..

[27] Roncoli C., Bekiaris-Liberis N., Papageorgiou M. Optimal lane-changing control at motorway bottlenecks. Proceedings of the 2016 IEEE 19th International Conference on Intelligent Transportation Systems (ITSC).

[28] Li Y., Xu C., Xing L., Wang W. (2017). Integrated cooperative adaptive cruise and variable speed limit controls for reducing rear-end collision risks near freeway bottlenecks based on micro-simulations. IEEE Trans. Intell. Transp. Syst..

[29] Xu H., Zhang Y., Cassandras C.G., Li L., Feng S. (2020). A bi-level cooperative driving strategy allowing lane changes. Transp. Res. Part C Emerg. Technol..

[30] Milanes V., Godoy J., Villagra J., Perez J. (2011). Automated On-Ramp Merging System for Congested Traffic Situations. IEEE Trans. Intell. Transp. Syst..

[31] Daganzo C.F., Laval J., Munoz J.C. (2002). Ten Strategies for Freeway Congestion Mitigation with Advanced Technologies. UC Berkeley: California Partners for Advanced Transportation Technology.

[32] Wang Y., Ma W., Henrickson K.C., Wang Y., Yang X. (2015). Dynamic Lane Assignment Approach for Freeway Weaving Segment Operation. Transp. Res. Rec. J. Transp. Res. Board..

[33] Zhao J., Ma W., Liu Y., Han K. (2016). Optimal operation of freeway weaving segment with combination of lane assignment and on-ramp signal control. Transp. A Transp. Sci..

[34] An X., Zhao J., Li P., Ma X. (2019). Effect of lane allocation on operational efficiency at weaving areas based on a cellular automaton model. IET Intell. Transp. Syst..

[35] Zheng Y., Ran B., Qu X., Zhang J., Lin Y. (2020). Cooperative Lane Changing Strategies to Improve Traffic Operation and Safety Nearby Freeway Off-Ramps in a Connected and Automated Vehicles Environment. IEEE Trans. Intell. Transp. Syst..

[36] Sun S., An X., Zhao J., Li P., Shao H. (2021). Modeling and Simulation of Lane-Changing Management Strategies at On-Ramp and Off-Ramp Pair Areas Based on Cellular Automaton. IEEE Access.

[37] Tajalli M., Niroumand R., Hajbabaie A. (2022). Distributed cooperative trajectory and lane changing optimization of connected automated vehicles: Freeway segments with lane drop. Transp. Res. Part C Emerg. Technol..

[38] Wang Y., Wang L., Guo J., Papamichail I., Papageorgiou M., Wang F.Y., Bertini R., Hua W., Yang Q. (2022). Ego-efficient lane changes of connected and automated vehicles with impacts on traffic flow. Transp. Res. Part C Emerg. Technol..

[39] Gazis D.C., Rothery H. (1961). Nonlinear Follow-the-Leader Models of Traffic Flow. Oper. Res..

[40] Gipps P.G. (1981). A Behavioural Car-Following Model for Computer Simulation. Transp. Res. Part B Methodol..

[41] Davis L.C. (2002). Analysis of Optimal Velocity Model with Explicit Delay. Phys. Rev. E.

[42] Jiang R., Wu Q., Zhu Z. (2001). Full velocity difference model for a car-following theory. Phys. Rev. E.

[43] Treiber M., Hennecke A., Helbing D. (2000). Congested Traffic States in Empirical Observations and Microscopic Simulations. Phys. Rev. E.

[44] Kesting A., Treiber M., Helbing D. (2010). Enhanced intelligent driver model to access the impact of driving strategies on traffic capacity. Philos. Trans. A Math Phys. Eng..

[45] Panov A.I., Yakovlev K.S., Suvorov R. (2018). Grid Path Planning with Deep Reinforcement Learning: Preliminary Results. Procedia Comput. Sci..

[46] Huang Z., Chu D., Wu C., Yi H. (2019). Path Planning and Cooperative Control for Automated Vehicle Platoon Using Hybrid Automata. IEEE Trans. Intell. Transp. Syst..

[47] You F., Zhang R., Lie G., Wang H., Wen H., Xu J. (2015). Trajectory planning and tracking control for autonomous lane change maneuver based on the cooperative vehicle infrastructure system. Expert Syst. Appl..

[48] Choi Y.G., Lim K.I., Kim J.H. Lane change and path planning of autonomous vehicles using GIS. Proceedings of the International Conference on Ubiquitous Robots & Ambient Intelligence.

